# Habitats Show More Impacts Than Host Species in Shaping Gut Microbiota of Sympatric Rodent Species in a Fragmented Forest

**DOI:** 10.3389/fmicb.2022.811990

**Published:** 2022-02-07

**Authors:** Yuwei Teng, Xifu Yang, Guoliang Li, Yunlong Zhu, Zhibin Zhang

**Affiliations:** ^1^State Key Laboratory of Integrated Management of Pest Insects and Rodents in Agriculture, Institute of Zoology, Chinese Academy of Sciences, Beijing, China; ^2^CAS Center for Excellence in Biotic Interactions, University of Chinese Academy of Sciences, Beijing, China

**Keywords:** forest succession, gut microbiota, 16S, habitat, host species, diet shift, rodents

## Abstract

Gut microbiota play a significant role for animals to adapt to the changing environment. Host species and habitats are key drivers in shaping the diversity and composition of the microbiota, but the determinants of composition of the sympatric host gut microbiome remain poorly understood within an ecosystem. In this study, we examined the effects of habitats of different succession stages and host species on the diversity and composition of fecal gut microbiota in four sympatric rodent species (*Apodemus draco*, *Leopoldamys edwardsi*, *Niviventer confucianus*, and *Niviventer fulvescens*) in a subtropical forest. We found, as compared to the differences between species, habitat types showed a much larger effect on the gut microbiota of rodents. Alpha diversity of the microbial community of *A. draco*, *N. fulvescens*, and *N. confucianus* was highest in farmland, followed by primary forest and shrubland, and lowest in secondary forest. Beta diversity of the three rodent species showed significant different among habitats. The alpha diversity of gut microbiota of *L. edwardsi* was significantly higher than those of *A. draco* and *N. confucianus*, and its beta diversity showed significant difference from *A. draco*. Our results suggested that gut microbiota were important for animals in responding to diet changes in different habitats under human disturbances.

## Introduction

Mammalian digestive systems harbor a complex microbial community, which is essential for their hosts to digest food, maintain health, and adapt to the changing environments (Clemente et al., 2012). Changes in gut microbiota are found to be closely related to metabolic dysfunction and diseases of humans, including cancer, diabetes, obesity, and cardiovascular disease (Li et al., 2016). Many factors, such as phylogeny, environment, and diet, could influence the diversity and composition of gut microbiota (Maynard et al., 2012; Tremaroli and Bäckhed, 2012). Elucidating the ecological and evolutionary processes in shaping the composition of host-associated microbial communities remains a major challenge (Costello et al., 2012; Foster et al., 2017).

Gut microbial communities generally cluster by host family of animals (Ley et al., 2008; Phillips et al., 2012; Groussin et al., 2017). Phylogeny of host animals can be a strong predictor of gut microbiota, which has been confirmed in a diverse range of taxa, including insects, birds, and mammals (Ochman et al., 2010; Nishida and Ochman, 2018; Amato et al., 2019; Knowles et al., 2019). Associations between multiple genomic regions and abundance of different microbial taxa have been identified in mice when fed with a controlled diet (Benson et al., 2010; McKnite et al., 2012) and in humans (Li et al., 2012; Smeekens et al., 2014). Mouse knockout experiments have identified genes involved in metabolism, immunity, and behavior that affect the gut microbiota (Spor et al., 2011).

Many previous studies have demonstrated that both short-term diet changes and long-term dietary shifts could strongly alter the composition of gut microbiota (Ley et al., 2008; Carmody et al., 2015; Li et al., 2019). Dietary changes of animals are common in different seasons and habitats, which may affect their gut microbiota. For example, Sun et al. (2016) found that the gut microbiota composition of the Tibetan macaque (*Macaca thibetana*) showed a significant response to the seasonal fluctuations of food resources. The diversity and composition of the microbial community of the black howler monkey (*Alouatta pigra*) varied with habitat degradation (Amato et al., 2013).

Although effects of both host species and habitat change on microbes have been fully investigated across large-scale environment gradients (Amato et al., 2019; Knowles et al., 2019; Youngblut et al., 2019; Huang et al., 2021), their distinct roles in an ecosystem for regulating gut microbiota of sympatric species are rarely explored. Sympatric species may share both distinct and similar food items in an ecosystem, which is determined by food availability or diversity in different habitats and their inherited digesting ability on specific food items. Gut microbiota are recognized as the second genomes of hosts and play a very significant role for animals to adapt to changing environments as symbionts. Food diversity and availability often change greatly in different habitats, which would have a greater impact on gut microbiota of sympatric species.

Forest fragmentation caused by human activities has been identified as the most important factor leading to the decline and loss of global species diversity (Noss et al., 1994; Bogaert et al., 2011). Forest fragmentation leads to differences in available plant species and a reduction in plant diversity (Benítez-Malvido and Martínez-Ramos, 2003). Plant communities provide habitats and food sources for various animals; therefore, the effects of forest fragmentation on plant communities may also cause changes in animal communities (da Fonseca and Robinson, 1990; Shenko et al., 2012). Many studies have observed changes in species composition due to natural succession in re-growing areas (Lohbeck et al., 2013; Whitehead et al., 2014; Martínez-Ramos et al., 2016). Our previous studies have found that the species richness and abundance of seeds and rodents varied greatly with stand age (Yang et al., 2018), but their consequences of forest succession in shaping the gut microbiota of rodent species have not been investigated.

Here, by using 16S ribosomal RNA gene sequencing, we explored the impacts of host species and habitats with dietary change caused by forest fragmentation on the diversity and composition of fecal gut microbiota of four sympatric rodent species (*Apodemus draco*, *Leopoldamys edwardsi*, *Niviventer confucianus*, and *Niviventer fulvescens*) in a subtropical forest. The four rodent species share very similar food items (Yang et al., 2018) in four habitats, we selected along a gradient of forest succession in the Dujiangyan region, Sichuan Province, China. We want to test the following three hypotheses: (1) The diversity and composition of gut microbiota should differ among different species of rodents; (2) The diversity of gut microbiota of rodents should be higher in primary or old forests with more diversified seeds than in young forests; and (3) Habitats would explain more variance of gut microbiota than host species because the sympatric rodent species share similar foods in the study region.

## Materials and Methods

### Study Site

The study was performed in the Banruosi Experimental Forest (altitude, 600–1,000 m) of Dujiangyan city (31°04′N-31°05′N, 103°42′E-103°42′E), Sichuan Province, China. The site lies in the middle of the subtropical zone, with a mean annual temperature of 15.2°C and annual precipitation of 1,200–1,800 mm. In the study site, the common rodent species include South China filed mice (*A. draco*, AD), Edward’s long-tailed rats (*L. edwardsi*, LE), Chestnut rats (*N. fulvescens*, NF), and Chinese white-bellied rats (*N. confucianus*, NC). These rodent species mainly feed on similar tree seeds, such as nuts and acorns (Yang et al., 2018).

Our study was conducted in 12 forest patches in the study site in the autumn of 2020. Most of the forests were cleared in the 1980–2005s, and subsequently, forest fragments were allowed to regrow on hilltops while flatter areas were maintained for cultivation or roads by local people. These forest patches were classified into four categories based on stand age and the degree of human disturbance: (1) Farmland (F), in which was mainly planted with *Cryptomeria fortune*, a popular local crop plant, (2) Primary forest (P), which has been preserved for 100 years because of the protection from the Banruosi Temple, (3) Shrubland (SH), and (4) Secondary forest (SE). Both shrubland and secondary forest had undergone extensive logging and destruction in the 1980–2005s and represented early or middle succession stages, respectively. Each of the four kinds of habitats had three replicate patches.

### Rodent Trapping and Sampling

We used wire live traps (30 cm × 13 cm × 12 cm), baited with fresh chestnuts to trap small rodents in the study site. We set a 4 × 10 trapping grid with an interval of 10 m in each plot by following Yang et al. (2018). Traps were placed at 15:00–17:00 h in the afternoon and were checked at 7:30–9:30 h in the next morning. Captured rodents were transported to field laboratory for identification of species and classification of sex. We also recorded body mass and reproductive status (females pregnant, lactating, or not; males with testes descended or not). Captured rodents were sacrificed by cervical dislocation and then dissected. The caecum was removed to collect fecal content, then kept frozen at −20°C for 6–10 days, and stored at −80°C in the laboratory until DNA extraction.

### 16S rRNA Gene Sequencing and Data Analysis

Total genome DNA of microbiota within the cecal contents was extracted using the cetyltrimethylammonium bromide (CTAB) method, the purity and concentration of DNA were determined by Gel electrophoresis and diluted to 1 ng/μl by sterile water. Then, 16S rRNA genes were amplified using specific primers with adapter sequences. Primers were set corresponding to the forward primer 341F (5′-CCTAYGGGRBGCASCAG-3′) and reverse primer 806R (5′-GGA CTACNNGGGTATCTAAT-3′), targeting the V3–V4 hypervariable 16S rRNA gene region. Sequencing libraries were generated using Illumina TruSeq DNA PCR-Free Library Preparation Kit (Illumina, United States), and index codes were added. All libraries were sequenced on an Illumina NovaSeq platform and then 250 bp paired-end reads were generated.

### Bioinformatics Processing

All analyses of the 16S rRNA gene sequences were performed using QIIME1.9.1 (Caporaso et al., 2010), USEARCH 10.0 (Edgar, 2010), and in-house scripts. We merged paired-end sequences using the method from FLASH (Magoč and Salzberg, 2011). Merged sequences were filtered by QIIME quality filters. After quality control, sequence data were processed through the denoising analysis pipeline UNOISE3 to infer amplicon sequence variants (ASVs). Chimeric sequences were identified and removed using USEARCH. Based on the high confidence 16S representative sequences, a feature table was generated by USEARCH. The taxonomy of the representative sequences was classified with the RDP (Cole et al., 2008) classifier. We calculated the alpha diversity with the ASV richness, Shannon index, and ACE index.

### Statistical Analyses

Statistical analyses were performed using R version 4.0.3 (R Core Team, 2020). The differences in three alpha diversity indices among groups were assessed by one-way ANOVA with Tukey’s *post-hoc* test. Significant differences in beta diversity between different groups were evaluated by permutational multivariate ANOVA (PERMANOVA), which employed the *adonis* function in the R package vegan with 999 permutations. The difference in beta diversity based on three metrics (Bray–Curtis dissimilarity, weighted and unweighted UniFrac distances) at the ASV level was assessed by the constrained principal coordinate analysis (CPCoA). Differences in relative abundances of taxonomic units among groups were tested by using Tukey’s *post-hoc* test. The linear discriminant analysis (LDA) Effect Size (LEfSe) method was used to assess differences in microbial communities using an LDA score threshold of 2 (Segata et al., 2011).

## Results

### Interspecific Variation of Gut Microbiota in Rodents

A total of 159 rodents were caught in 12 forest patches in the study site from September to October 2020, including *A. draco* (*n* = 70), *N. fulvescens* (*n* = 41), *N. confucianus* (*n* = 40), and *L. edwardsi* (*n* = 8; Supplementary Table S1). We only selected four dominant species (*A. draco*, *N. fulvescens*, *N. confucianus*, and *L. edwardsi*) for analysis due to the insufficient number of other species.

**Figure 1:**
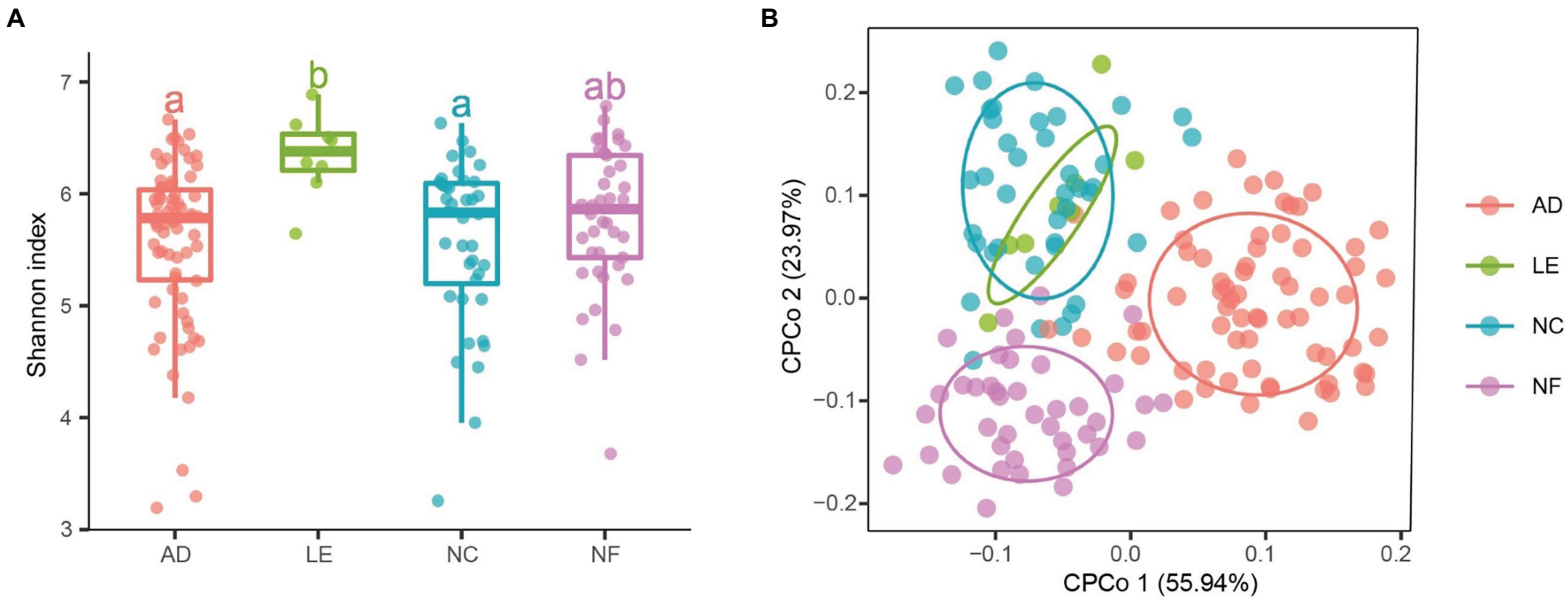
Variation of gut microbial diversity of caecum fecal samples between four rodent species. **(A)** Alpha diversity (Shannon index). Different letters represent statistical significance (*p* < 0.05). **(B)** Beta diversity comparisons of the gut microbiota of caecum fecal samples between the four rodent species. The first two axes are shown with constrained principal coordinate analysis (CPCoA) based on the Bray–Curtis dissimilarity matrix at the amplicon sequence variant (ASV) level. AD, *Apodemus draco*. LE, *Leopoldamys edwardsi*. NC, *Niviventer confucianus*. NF, *Niviventer fulvescens*.

Alpha diversity analysis indicated that there were significant differences in richness, Shannon index, and ACE index between rodent species (Figure 1A; Supplementary Figure S1). The three diversity indices of *L. edwardsi* were significantly larger than in *A. draco* (richness, *p* = 0.002; ACE, *p* = 0.008; and Shannon, *p* = 0.018) and *N. confucianus* (richness, *p* = 0.017; ACE, *p* = 0.042; and Shannon, *p* = 0.026). Beta diversity analysis using PERMANOVA revealed that there was a significant difference in gut microbiota community between *A. draco* and *L. edwardsi* (adonis permutation test, *F* = 1.700, *p* = 0.007; Figure 1B).

**Figure 2:**
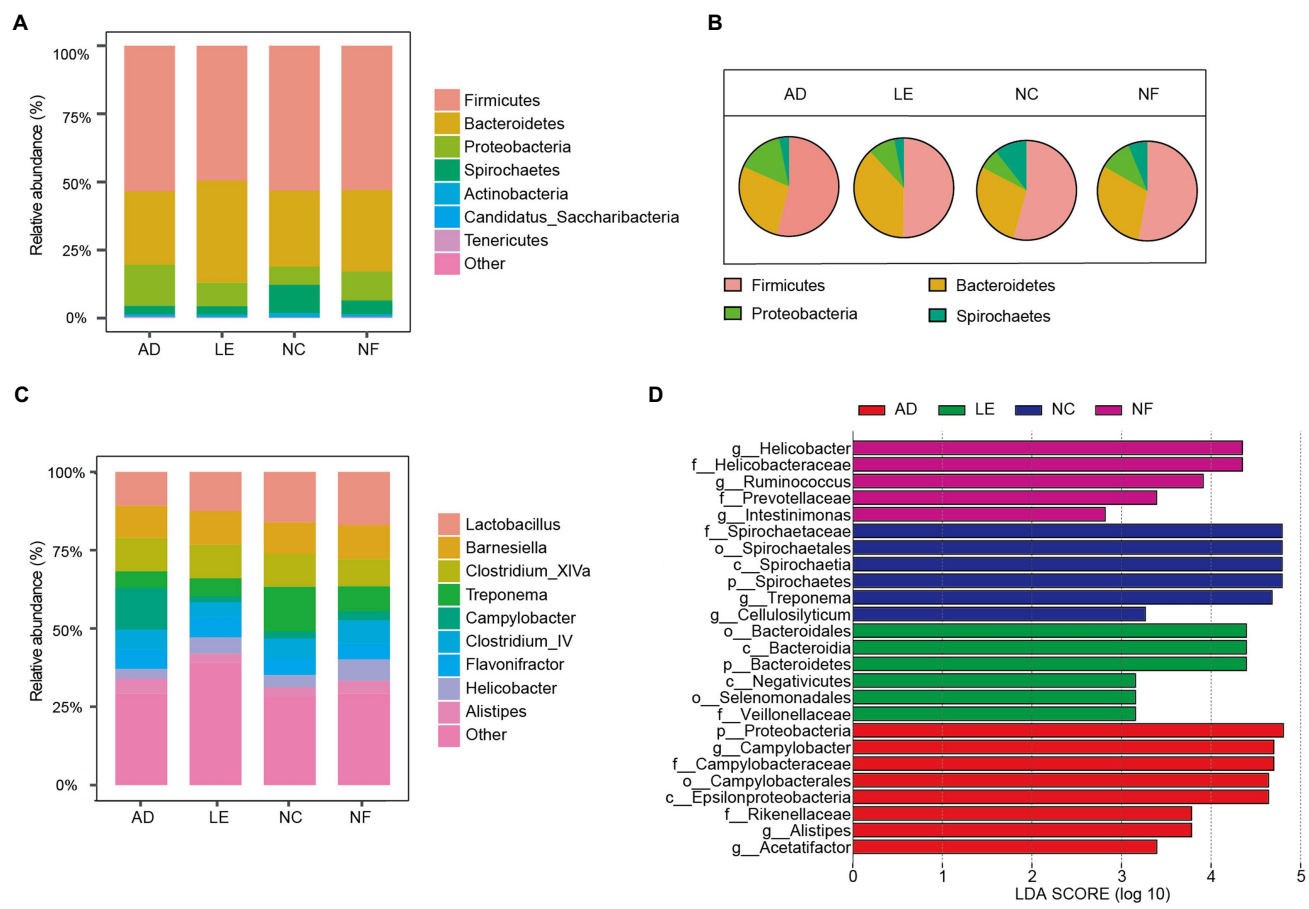
Variation of gut microbial composition between four rodent species. **(A)** Abundance represented as the proportions of ASVs classified at the phylum rank. **(B)** Pie chart of four main phyla in four rodent species. **(C)** Abundance represented as the proportions of ASVs classified at the genus rank. **(D)** Differential bacterial taxa selected by linear discriminant analysis (LDA) Effect Size (LEfSe) analysis with LDA score >2 in gut microbiota community of four species.

The most abundant bacterial phylum of the four rodent species was Firmicutes (mean = 52.91%), Bacteroidetes (28.54%), Proteobacteria (11.20%), and Spirochaetes (5.69%). The main components of each species at the phylum level were visualized in Figure 2A. The pie chart showed that there were significant differences in the main phylum of the four species (Figure 2B). The proportion of Bacteroidetes in *L. edwardsi* was significantly higher than in *A. draco* (*L. edwardsi* vs. *A. draco*, *p* = 0.024), Proteobacteria in *A. draco* were significantly higher than in *N. confucianus* (*A. draco* vs. *N. confucianus*, *p* < 0.001), and Spirochaetes in *N. confucianus* and *N. fulvescens* was significantly higher than in *A. draco* (*N. confucianus* vs. *A. draco*, *p* < 0.001; *N. fulvescens* vs. *A. draco*, *p* = 0.01; Supplementary Figure S2). At the genus level, the gut microbiota of the four species were dominated by *Lactobacillus* (7.84%), *Barnesiella* (6.01%), *Clostridium_XlVa* (5.75%), *Treponema* (4.98%), *Campylobacter* (3.87%), *Clostridium_IV* (3.60%), *Flavonifractor* (3.35%), *Helicobacter* (2.42%), and *Alistipes* (2.20%; Figure 2C).

The LEfSe results revealed that there were significant differences in the compositions of the gut microbial community among four rodent species (Figure 2D). At the phylum level, Proteobacteria were enriched in *A. draco*, and Bacteroidetes was enriched in *L. edwardsi*, whereas Spirochaetes was enriched in *N. confucianus*. At the family level, Campylobacteraceae and Rikenellaceae were enriched in *A. draco*, Veillonellaceae was enriched in *L. edwardsi*, and Spirochaetaceae was enriched in *N. confucianus*, whereas Helicobacteraceae and Prevotellaceae were enriched in *N. fulvescens*. At the genus level, *Campylobacter*, *Alistipes*, and *Acetatifactor* were enriched in *A. draco*, and *Treponema* and *Cellulosilyticum* were enriched in *N. confucianus*, whereas *Helicobacter*, *Ruminococcus*, and *Intestinimonas* were enriched in *N. fulvescens*.

### Effect of Habitats on Gut Microbiota in Rodents

**Figure 3:**
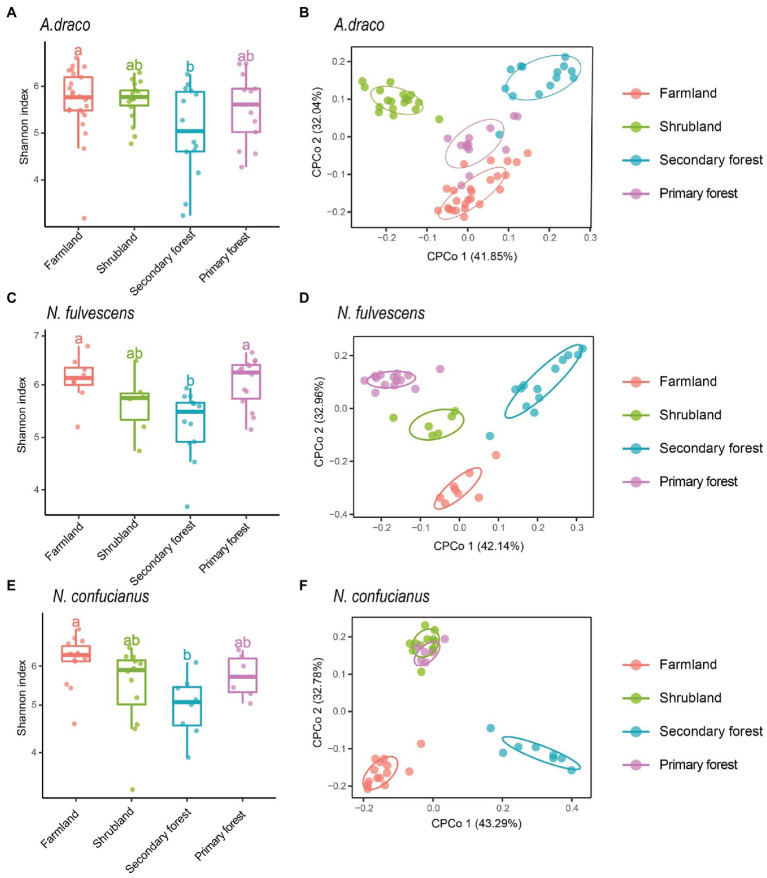
Variation of gut microbial diversity of three rodent species in four different habitats. Alpha diversity (Shannon index) of bacterial communities of *Apodemus draco* **(A)**, *Niviventer fulvescens* **(C)**, and *Niviventer confucianus* **(E)** across four kinds of habitats. Different letters represent statistical significance (*p* < 0.05). Beta diversity comparisons of the gut microbiota of *A. draco* **(B)**, *N. fulvescens* **(D)**, and *N. confucianus* **(F)** in four habitat types. The first two axes are shown with constrained CPCoA based on the Bray–Curtis dissimilarity matrix at the ASV level.

We only selected three species (*A. draco*, *N. fulvescens*, and *N. confucianus*) to analyze the effects of habitats on gut microbiota of rodents due to the insufficient number of *L. edwardsi* in each succession stage. Habitats showed a significant association with the alpha diversity of gut microbiota of the three rodent species. For the three rodent species, the Shannon index of gut microbiota had a consistent trend in different habitats, that is, the highest in farmland, followed by primary forest and shrubland, and the lowest in secondary forest; the Shannon index in farmland was significantly higher than that in secondary forest (*A. draco*, *p* = 0.039; *N. fulvescens*, *p* = 0.007; and *N. confucianus*, *p* = 0.011; Figure 3; Supplementary Table S2). The other two diversity indices (richness and ACE index) of the three rodent species also showed a similar trend to Shannon’s index at different succession stages (Supplementary Figure S3; Supplementary Table S2).

Beta diversity analysis using the CPCoA revealed that habitat type had a significant effect on the gut microbiota community of *A. draco* (*F* = 2.624, *p* = 0.001; 9.94% of variance explained; Figure 3B), *N. fulvescens* (*F* = 1.495, *p* = 0.002; 12.8% of variance explained; Figure 3D), and *N. confucianus* (*F* = 1.761, *p* = 0.001; 14.4% of variance explained; Figure 3F). These results showed a significant association between forest succession stage and composition of gut microbiota of all rodent species.

**Figure 4:**
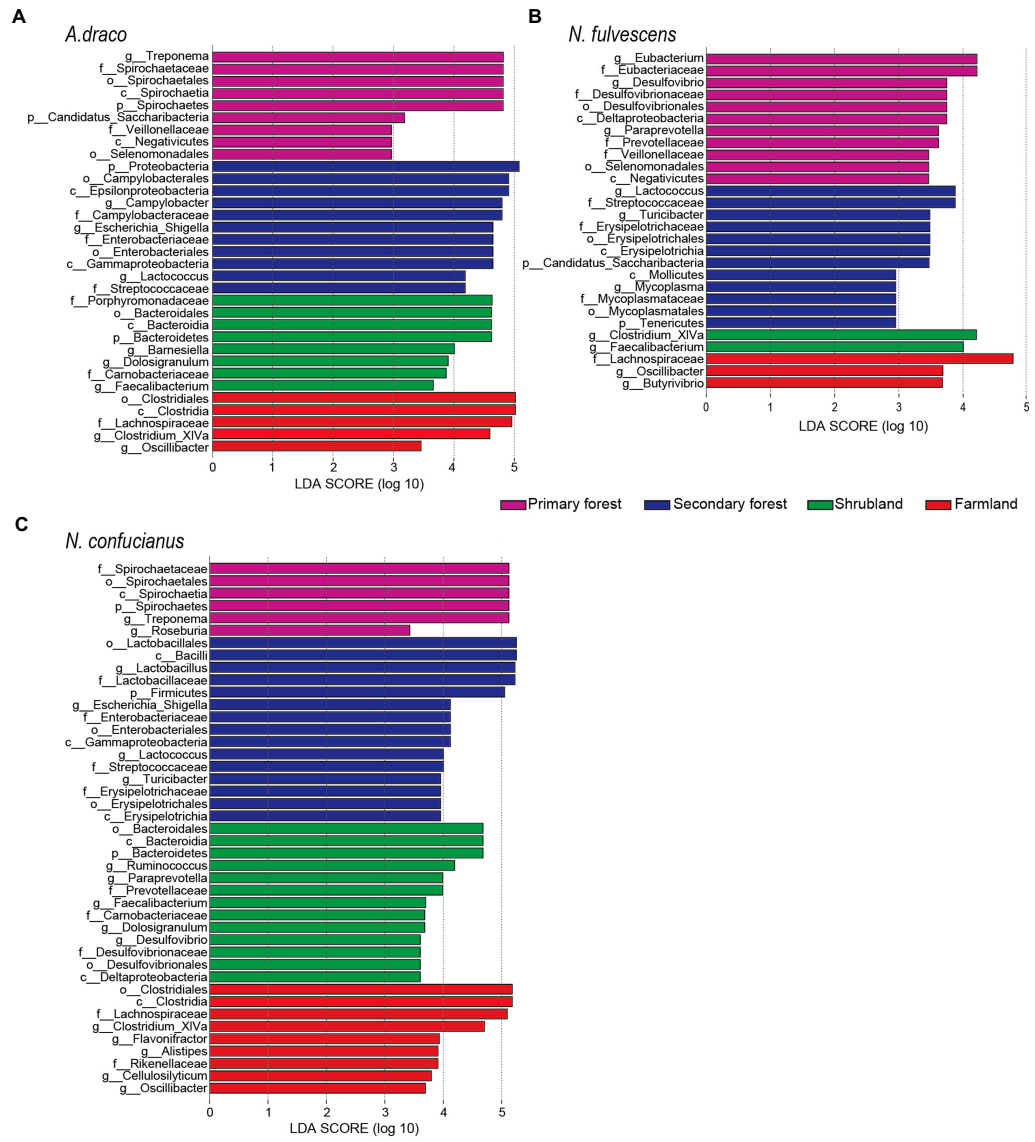
Different bacterial taxa selected by LEfSe analysis with LDA score > 2 in gut microbiota community. Differential bacterial taxa of *Apodemus draco* **(A)**, *Niviventer fulvescens* **(B)**, and *Niviventer confucianus* **(C)** in four habitat types.

The LEfSe results revealed that there were significant differences in the gut microbiota of *A. draco* among four habitat types. At the phylum level, Bacteroidetes was enriched in shrubland, Proteobacteria were enriched in secondary forest, and Spirochaetes and Candidatus_Saccharibacteria were enriched in primary forest. At the family level, Lachnospiraceae was enriched in farmland; Porphyromonadaceae and Carnobacteriaceae were enriched in shrubland; Campylobacteraceae, Enterobacteriaceae, and Streptococcaceae were enriched in the secondary forest; and Spirochaetaceae and Veillonellaceae were enriched in primary forest. At the genus level, *Clostridium_XIVa* and *Oscillibacter* were enriched in farmland; *Barnesiella*, *Dolosigranulum*, and *Faecalibacterium* were enriched in shrubland; *Campylobacter*, *Escherichia_Shigella*, and *Lactococcus* were enriched in the secondary forest; and *Treponema* was enriched in primary forest (Figure 4A).

The LEfSe results revealed that there were significant differences in the gut microbiota of *N. fulvescens* among four habitat types. At the phylum level, Candidatus_Saccharibacteria and Tenericutes were enriched in secondary forest. At the family level, Lachnospiraceae was enriched in farmland; Streptococcaceae, Erysipelotrichaceae, and Mycoplasmataceae were enriched in the secondary forest; and Eubacteriaceae, Desulfovibrionaceae, Prevotellaceae, and Veillonellaceae were enriched in primary forest. At the genus level, *Oscillibacter* and *Butyrivibrio* were enriched in farmland; *Clostriduum_XIVa* and *Faecalibacterium* were enriched in shrubland; *Lactococcus*, *Turicibacter*, and *Mycoplasma* were enriched in the secondary forest; and *Eubacterium*, *Desulfovibrio*, and *Paraprevotella* were enriched in primary forest (Figure 4B).

The LEfSe results revealed that there were significant differences in the gut microbiota of *N. confucianus* among four habitat types. At the phylum level, Bacteroidetes was enriched in shrubland, Firmicutes was enriched in secondary forest, and Spirochaetes was enriched in primary forest. At the family level, Lachnospiraceae and Rikenellaceae were enriched in farmland; Prevotellaceae, Carnobacteriaceae, and Desulfovibrionaceae were enriched in shrubland; four families (Lactobacillaceae, Enterobacteriaceae, Streptococcaceae, and Erysipelotrichaceae) were enriched in the secondary forest; and Spirochaetaceae was enriched in primary forest. At the genus level, five genera (*Clostridium_XIVa*, *Alistipes*, *Flavonifractor*, *Cellulosilyticum*, and *Oscillibacter*) were enriched in farmland; five genera (*Ruminococcus*, *Paraprevotella*, *Dolosigranulum*, *Desulfovibrio*, and *Faecalibacterium*) were enriched in shrubland; four genera (*Lactobacillus*, *Escherichia_Shigella*, *Lactococcus*, and *Turicibacter*) were enriched in the secondary forest; and *Treponema* and *Roseburia* were enriched in primary forest (Figure 4C).

### Effect of Host Species and Habitats on Gut Microbiota in Rodents

**Figure 5:**
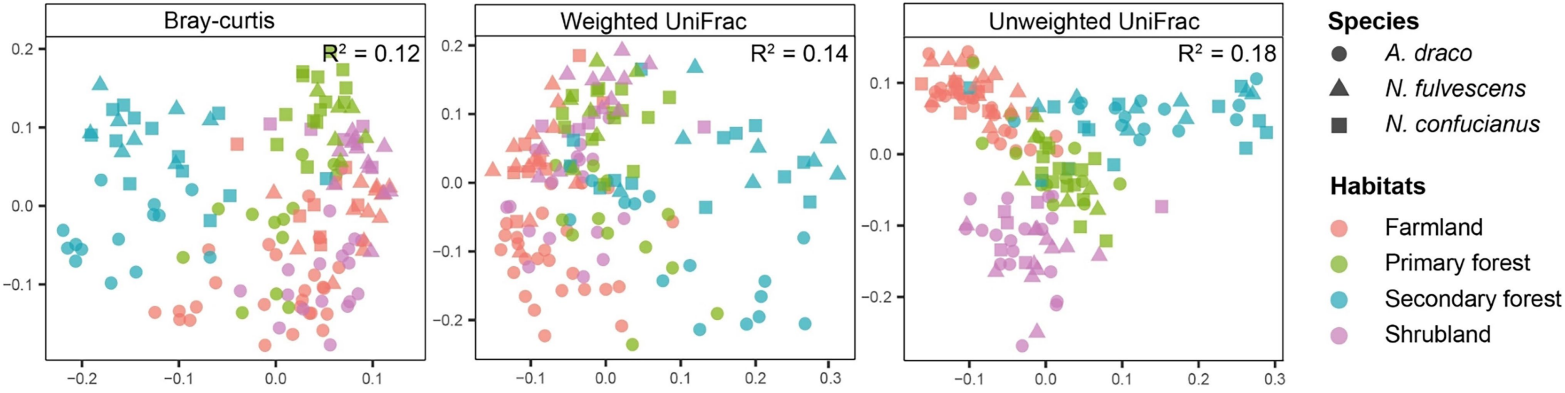
Constrained principal coordinates analysis plots showing how clustering of samples by habitats varies across three dissimilarity metrics that differ in their sensitivity to the phylogenetic relatedness and abundance of bacterial sequence variants. *R^2^* values from permutational multivariate ANOVAs (PERMANOVAs) testing the effect of habitats are shown on each plot.

Constrained principal coordinates analysis showed the gut microbiota of rodents relatively clear sample cluster by habitats, but less so by host species (Figure 5). Results of PERMANOVAs testing also showed that the effect of habitats on gut microbiota in rodents was more explanatory than that between sympatric species (Table 1). These results indicated that habitats under different forest succession showed more impacts than host species in shaping gut microbial communities of sympatric rodent species.

**Table 1:** Results of PERMANOVAs testing for the effect of habitats and host species on gut microbiota in rodents, using three different dissimilarity metrics.

Dissimilarity metric	Habitats			Host species		
	*F*	*p*	*R^2^*	*F*	*p*	*R^2^*
Bray-Curtis	10.66	<0.001	0.18	2.81	<0.001	0.03
Weighted UniFrac	8.02	<0.001	0.14	4.53	<0.001	0.05
Unweighted UniFrac	6.99	<0.001	0.12	5.08	<0.001	0.06

PERMANOVAs were run using the adonis function in R package vegan, using 999 permutations.

## Discussion

It is known that host species and habitats could significantly affect the gut microbiota of small mammals across a broad scope of taxa and environmental gradients, but it is unclear how the gut microbiota of sympatric species responds to habitat changes caused by forest fragmentation. In this study, we found both host species and habitat type contributed to the variance of gut microbiota of rodents. However, the difference of gut microbiota between four sympatric rodent species was relatively small, while the difference between four habitats was much larger, suggesting that habitats with dietary change may be more important in shaping the microbiota community of sympatric rodents. Three rodent species (*A. draco*, *N. fulvescens*, and *N. confucianus*) showed significant difference in beta diversity of gut microbiota between habitats, and their alpha diversities were highest in farmland, followed by primary forest and shrubland, and lowest in secondary forest. *L. edwardsi* showed significant higher alpha diversity of gut microbiota than those of *A. draco* and *N. confucianus*, and significant difference of beta diversity with *A. draco*. These differences may be related to differences in food diversity between species or habitats.

### Interspecific Variation on Gut Microbiota Between Rodent Species

Gut microbiota are likely shaped by diverse host factors, such as behavioral (i.e., social contact patterns), heritable (e.g., evolutionary history and genetics), and environmental (e.g., diet and geography; Kasper, 2014; Perofsky et al., 2019; Li et al., 2021; Wan et al., 2021). In this study, our results showed that the diversity of gut microbiota in *L. edwardsi* was significantly different from those of *A. draco* and *N. confucianus*, while the interspecific differences of the other three rodent species were not significant, which support our first hypothesis. This is likely because the body size of *L. edwardsi* is the largest one (Mean ± SE, 309.19 ± 43.65 g), which could hoard and eat seeds of most tree species including large- and small-sized seeds and seeds with both hard- and soft-seed coats, while the body size of the other species was relatively small (*A. draco*, 28.87 ± 0.85 g; *N. confucianus*, 83.10 ± 3.21 g; and *N. fulvescens*, 69.84 ± 2.86 g) and could only eat small-sized or soft-coated seeds (Chang and Zhang, 2014; Yang et al., 2018). This well explained the observation that alpha diversity of *L. edwardsi* was significantly larger than *A. draco* and *N. confucianus*. All four rodent species prefer to feed on seeds of *Quercus serrata* with higher tannin content (Yang et al., 2018). *Lactobacillus*, the bacteria that can produce tannin-degrading enzymes (Osawa et al., 2006), may account for a high proportion of the four rodents (Figure 2C).

We found that the composition of gut microbiota of *A. draco*, *L. edwardsi*, *N. confucianus*, and *N. fulvescens* was significantly different at different taxonomic levels (Figure 2), which support our first hypothesis. At the phylum level, the gut microbiota of the four rodents were dominated by Firmicutes and Bacteroidetes, which were consistent with other herbivores, such as pikas (Li et al., 2018), horses (Costa et al., 2012), and rabbits (Velasco-Galilea et al., 2018). The abundance of Proteobacteria in *A. draco* tended to be higher than that of *L. edwardsi*, *N. confucianus*, and *N. fulvescens*. High-fat and high-fructose diet in mice have been proven to cause a rise in Proteobacteria (Jeong et al., 2019; Vasques-Monteiro et al., 2021). It is known that *A. draco* prefers to eat high-fat seeds, such as oil teas, which contains high fat (Gu et al., 2021).

In addition, our results suggested that many of the species found in these rodents’ microbiome could be potentially zoonotic agents. High abundance of Proteobacteria which has been found in *A. draco* considered to be associated with dysbiosis in hosts with metabolic or inflammatory disorders (Mukhopadhya et al., 2012). The abundance of Spirochaetes in *N. confucianus* tended to be higher than that of *L. edwardsi*, *A. draco*, and *N. fulvescens*. The phylum Spirochaetes consists of a large group of motile bacteria that are widespread in the environment and are highly prevalent disease-causing agents. The Spirochaetes species cause many important diseases including syphilis and Lyme disease (Gupta et al., 2013). At the genus level, *Campylobacter* and *Alistipes* were enriched in *A. draco*. *Campylobacter* has been recognized as an important human and mammalian pathogen, many of which are considered to be pathogens of gastroenteritis of etiology (Man et al., 2010). *Alistipes* is a relatively new genus of bacteria, believed to be closely related to ecological disorders (dysbiosis) and diseases (Parker et al., 2020). *Treponema* was enriched in *N. confucianus*. Some bacteria from *Treponema* are thought to be related to mammalian diseases (Paster and Canale-Parola, 1985; McKenna et al., 2008). *Helicobacter* was enriched in *N. fulvescens*. *Helicobacter* is believed to have pathogenic and pro-inflammatory effects (Xie et al., 2020). Besides, *A. draco*, *N. fulvescens*, and *N. confucianus* are relatively abundant, which may explain they had more disease-related microbes because high density would increase the prevalence of pathogens (Keesing et al., 2006).

### Effect of Habitats on Gut Microbiota of Rodent Species

Multiple environmental factors, including diet, geography, and living conditions, can influence microbial communities. Diet is one of the most important environmental factors that affect the composition of gut microbiota (Muegge et al., 2011; Carmody et al., 2015; Li et al., 2019). Previous studies on animals and humans have shown that diet strongly influences the composition of the gut microbiota (David et al., 2014; Carmody et al., 2015; Sonnenburg et al., 2016). Studies of wildlife have also reported strong environmental impacts, including differences in seasons and habitats (Amato et al., 2013; Maurice et al., 2015; Ren et al., 2017). In this study, we found significant differences in the diversity and composition of the gut microbiota of *A. draco*, *N. fulvescens*, and *N. confucianus* in different habitats. For *A. draco*, *N. fulvescens*, and *N. confucianus*, we observed that *Lactococcus* in secondary forest, *Faecalibacterium* in shrubland, and *Oscillibacter* and Lachnospiraceae in farmland were higher than in other habitats. This paper did not consider the role of seasonal factors, because we only analyzed the data of one season (i.e., autumn), and the influence of seasonal factors on the gut microbiota of small mammals will be considered in the subsequent study.

Biodiversity is an important aspect of ecosystem function and is essential to improve the resilience of macro-ecosystems. There is a view that, like all healthy ecosystems, the abundance of microbial species is a characteristic of the gut microbiota of healthy individuals (Heiman and Greenway, 2016). The high diversity of the gut microbiota may make the microbial ecosystem more resistant to external disturbances (Lozupone et al., 2012). Conversely, the loss of microbial diversity is usually associated with several disease states (Ott et al., 2004; Ley et al., 2006). In our study, we found that the alpha diversity of microbial community of rodents was highest in farmland, followed by primary forest and shrubland, and lowest in secondary forest, which does not support our second hypothesis (Figure 2; Supplementary Figure S3; Supplementary Table S2). Our previous study found that seed richness increased as the succession stage increased by analyzing the collected fallen seeds from early September to late December when seeds became mature (Yang et al., 2018). The primary (old) forest had an obvious more diversified seed species than the shrubland and secondary forest; thus, it is plausible that the high diversity of microbes was likely associated with the high diversity of seed species in different habitats. Notably, the microbial diversity of rodents in farmland is significantly higher than that in other forests, which is unexpected because the seed species in farmland should be smaller. The plausible explanation is that the edge effects might have impacts on species diversity and composition, community dynamics, and ecosystem functions (Laurance et al., 2007). The farmlands we selected are relatively small patches and closely adjacent to the surrounding primary forests and cropland, which might have increased the food diversity.

We also found that the composition of gut microbiota of *A. draco*, *N. fulvescens*, and *N. confucianus* changed continuously with stand age. The abundance of *Lactococcus* in the microbial community of *A. draco*, *N. fulvescens*, and *N. confucianus* living in secondary forests was significantly higher than that of other forests. It was found that the probiotic *Lactococcus*, a Streptococcaceae family, was contained in the gut of obese mice higher than that of lean mice when studying the link between obesity and the gut microbiota (Jiao et al., 2018). We speculated that this may be related to the different nutritional values and abundance of the main plant seeds distributed in different forests (Yang et al., 2018). Bacteria from *Lactococcus* can produce tannin-degrading enzymes (Mukherjee et al., 2014). Seeds of *Quercus serrata* and *Q. variabilis* with high tannin content account for a high proportion in the secondary forest (Yang et al., 2018). The abundance of *Faecalibacterium* in the microbial community of *A. draco*, *N. fulvescens*, and *N. confucianus* living in the shrubland was significantly higher than that of other forests. *Faecalibacterium*, as an important butyrate-producing bacteria, was seen to have higher abundances in all healthy subjects (Anand et al., 2016). The abundance of Lachnospiraceae and *Oscillibacter* in the microbial community of *A. draco*, *N. fulvescens*, and *N. confucianus* living in the farmland was significantly higher than that of other forests. The level of Lachnospiraceae was correlated positively with leptin level and negatively with energy consumption (Méndez-Salazar et al., 2018). *Oscillibacter* is increased by high saturated fat, high-resistant starch, and carbohydrate weight loss diets in mice (Walker et al., 2011; Lam et al., 2012) and is considered to have pathogenic and pro-inflammatory effects (Xue et al., 2020). In farmland, people or livestock may carry *Oscillibacter* and spread to rodents.

### Effect of Host Species and Habitats on Gut Microbiota in Rodents

The relative importance of habitats and host species in shaping the gut microbiota has been a major topic of debate (Spor et al., 2011; Carmody et al., 2015; Rothschild et al., 2018; Knowles et al., 2019). Within-species studies often report relatively weak phylogenetic signals compared to habitat effects (Carmody et al., 2015; Rothschild et al., 2018), whereas a cross-species comparisons have tended to emphasize host species, wherein gut microbiota similarity among species mirror the host species (Brucker and Bordenstein, 2012; Brooks et al., 2016). Our results showed that gut microbiota of sympatric rodent species were shaped more strongly by habitats than host species (Figure 5; Table 1), supporting our third hypothesis and previous findings (Carmody et al., 2015; Rothschild et al., 2018). Sympatric species living in the same environment will generally share similar food items, which may lead to a greater impact of habitats on the gut microbiota of sympatric rodent species than host species effects.

In summary, forest fragmentation can affect the abundance of plant seeds and animals, which may result in dietary shifts and then affect the diversity of gut microbiota of sympatric rodents. Our results showed that both habitats and host species had significant effects on the gut microbiota of sympatric rodents, but habitats explain more variance of gut microbiota of rodents than host species. Our results demonstrate that the process of forest succession caused by human activities plays an essential role in shaping the gut microbiota of sympatric rodents in fragmented habitats. Future research should focus on how dietary shifts result in changes in gut microbiota, and the significance of gut microbes in helping animals to adapt to the changing environments.

## Data Availability Statement

The original 16S rRNA sequence data are available in the NCBI Sequence Read Archive under accession PRJNA791199.

## Ethics Statement

The animal study was reviewed and approved by the Animal Welfare Ethics Committee of the Institute of Zoology, Chinese Academy of Sciences.

## Author Contributions

ZZ, YT, and XY designed the study. YT, XY, and YZ collected the data, which were analyzed by YT and GL. YT, XY, GL, and ZZ drafted the manuscript. All authors contributed to the article and approved the submitted version.

## Funding

This study was supported by the Strategic Priority Research Program and the key project of Chinese Academy of Sciences (XDB11050300), the National Key Research and Development Program (2017YFC0503802), and the National Natural Science Foundation of China (32001123 and 32070460).

## Conflict of Interest

The authors declare that the research was conducted in the absence of any commercial or financial relationships that could be construed as a potential conflict of interest.

## Publisher’s Note

All claims expressed in this article are solely those of the authors and do not necessarily represent those of their affiliated organizations, or those of the publisher, the editors and the reviewers. Any product that may be evaluated in this article, or claim that may be made by its manufacturer, is not guaranteed or endorsed by the publisher.
