# Supplementary material for: Habitats Show More Impacts Than Host Species in Shaping Gut Microbiota of Sympatric Rodent Species in a Fragmented Forest
Source: Front Microbiol. 2022 Feb 7;13:811990. doi: 10.3389/fmicb.2022.811990 (PMC8859092; doi:10.3389/fmicb.2022.811990)
Supplement: Supplementary file 1 [file Data_Sheet_1.docx]

Supplementary Material

# Supplementary Figures and Tables

## Supplementary Figures


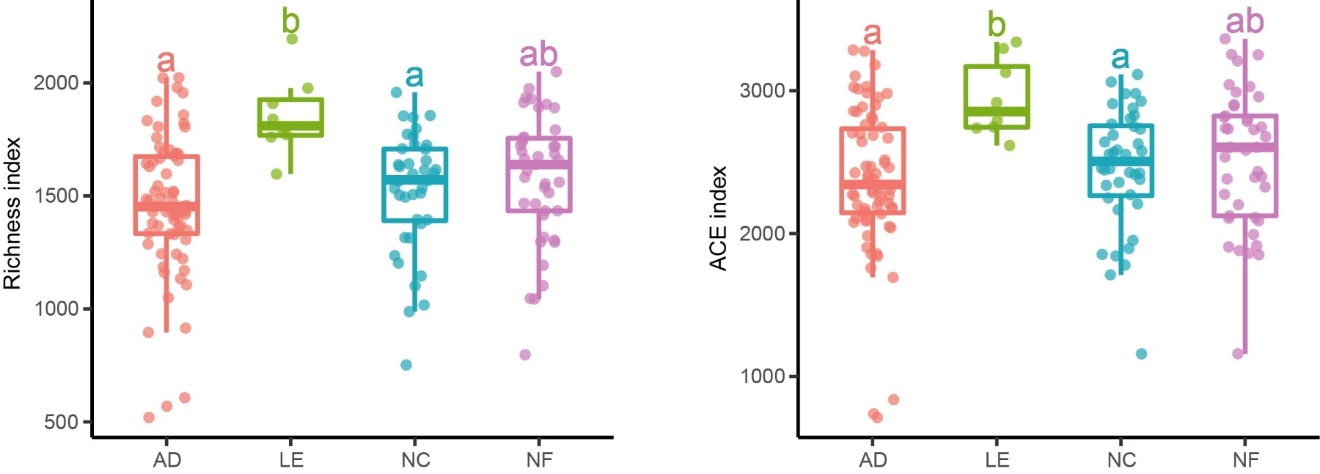


**Supplementary Figure 1.** Alpha diversity (richness and ACE index) of bacterial communities across four rodents. Different letters represent statistical significance (*p* < 0.05).


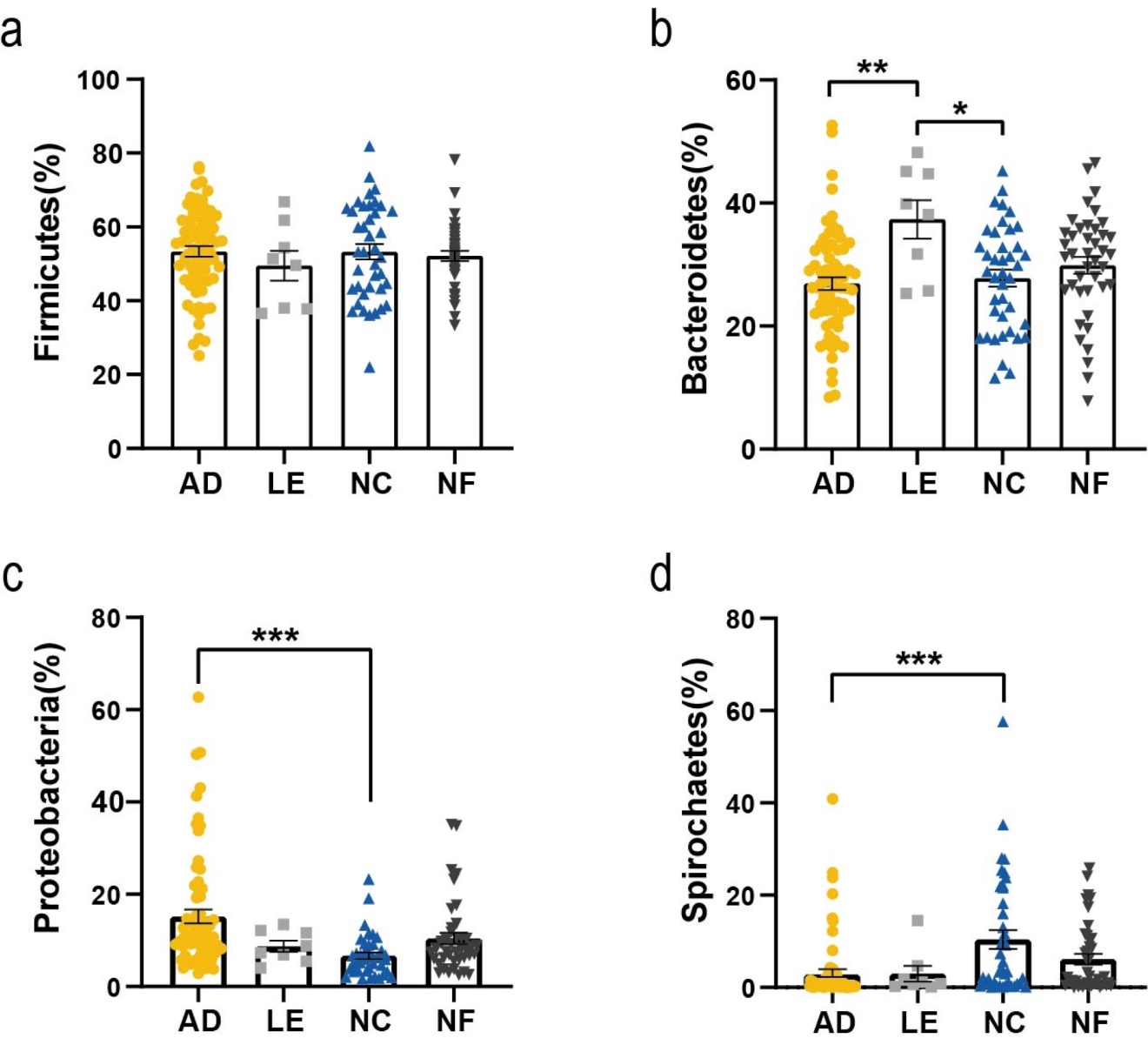


**Supplementary Figure 2.** Abundance represented as the proportions of ASVs classified at the phylum rank.


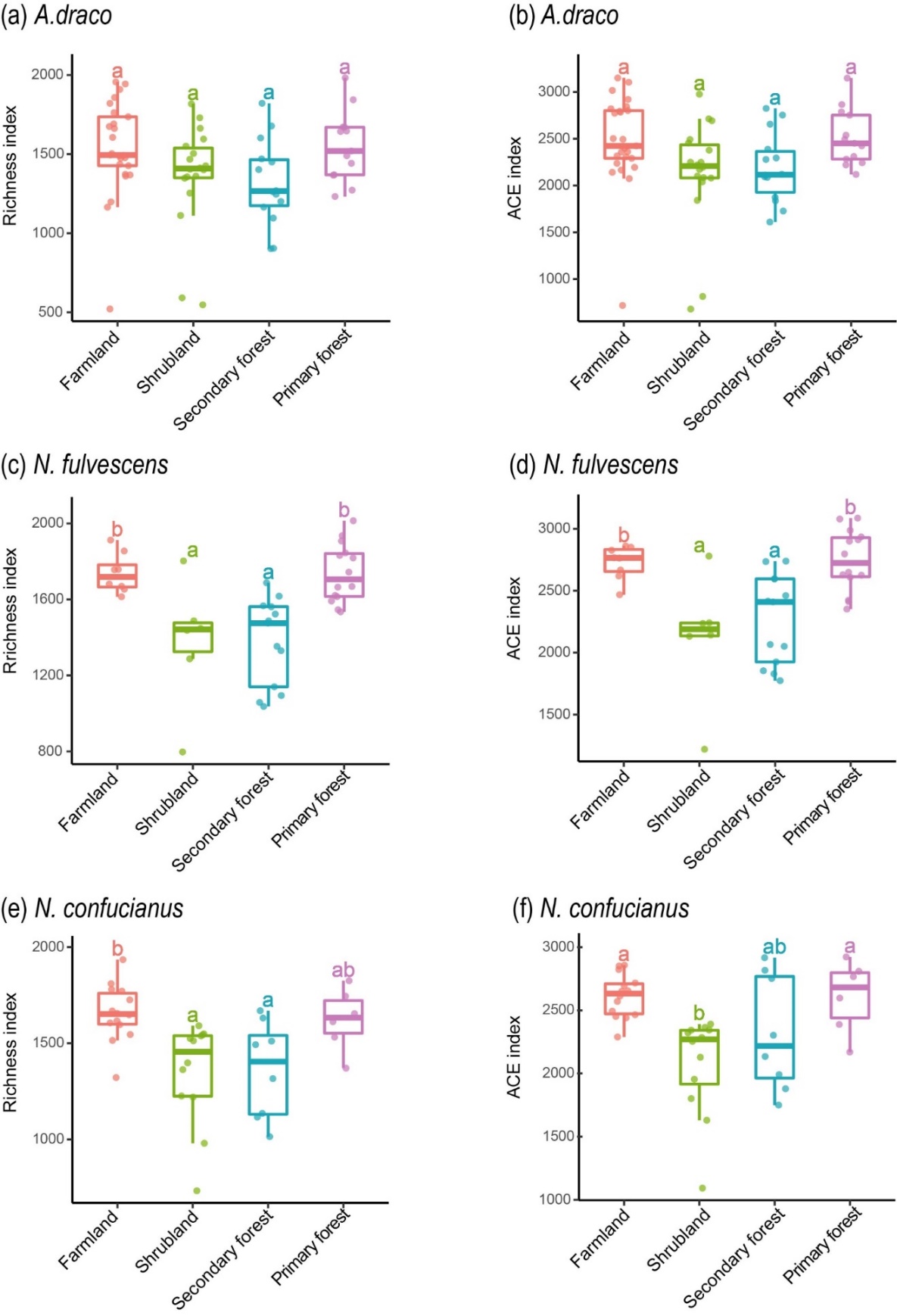


**Supplementary Figure 3.** Variation of alpha diversity of three rodents in four different succession stages. Shannon index of bacterial communities of *A. draco* (a), *N. fulvescens* (c), and *N. confucianus* (e) across four kinds of habitats. ACE index of bacterial communities of *A. draco* (b), *N. fulvescens* (d), and *N. confucianus* (f) across four kinds of habitats. Different letters represent statistical significance (*p* < 0.05).

## Supplementary Tables.

**Supplementary Table 1.** Composition of rodents captured at each habitat for which the gut microbiota was characterized in the Dujiangyan region.

| Species | Habitats | | | |
| --- | --- | --- | --- | --- |
|  | Farmland | Shrubland | Secondary forest | Primary forest |
| *A. draco* | 25 | 18 | 14 | 13 |
| *N. fulvescens* | 8 | 6 | 13 | 14 |
| *N. confucianus* | 14 | 12 | 8 | 6 |
| *L. edwardsi* | 3 | 1 | 1 | 3 |

**Supplementary Table 2.** The alpha diversity of gut microbiota of three rodents in four different habitats.

| Species | Habitats | Richness | ACE index | Shannon index |
| --- | --- | --- | --- | --- |
| *A. draco* | Farmland | 1535.96±60.91^a^ | 2480.16±97.48^a^ | 5.69±0.14^a^ |
|  | Shrubland | 1363.50±78.69^a^ | 2135.61±135.80^a^ | 5.69±0.10^ab^ |
|  | Secondary forest | 1319.36±72.27^a^ | 2190.16±99.44^a^ | 5.04±0.26^b^ |
|  | Primary forest | 1549.92±61.03^a^ | 2510.78±83.25^a^ | 5.52±0.20^ab^ |
|  | *F* | 2.678 | 2.995 | 2.942 |
|  | *p*-value | 0.054 | 0.037 | 0.039 |
| *N. fulvescens* | Farmland | 1737.38±36.63^b^ | 2728.09±47.97^b^ | 6.10±0.16^a^ |
|  | Shrubland | 1376.33±134.98^a^ | 2125.18±206.01^a^ | 5.63±0.24^ab^ |
|  | Secondary forest | 1379.23±63.21^a^ | 2265.44±99.63^a^ | 5.27±0.17^b^ |
|  | Primary forest | 1738.43±41.23^b^ | 2742.45±66.40^b^ | 6.03±0.12^a^ |
|  | *F* | 10.467 | 9.192 | 6.056 |
|  | *p*-value | 0.000 | 0.000 | 0.002 |
| *N. confucianus* | Farmland | 1656.93±39.66^b^ | 2612.09±45.67^a^ | 2612.09±45.67^a^ |
|  | Shrubland | 1348.33±76.88^a^ | 2075.67±114.16^b^ | 2075.67±114.16^ab^ |
|  | Secondary forest | 1360.75±88.72^a^ | 2317.68±161.00^ab^ | 2317.68±161.00^b^ |
|  | Primary forest | 1623.17±65.46^ab^ | 2608.57±116.26^a^ | 2608.57±116.26^ab^ |
|  | *F* | 6.36 | 6.685 | 3.963 |
|  | *p*-value | 0.001 | 0.001 | 0.015 |

Different superscripted letters indicate significant differences among groups (one-way ANOVA, Tukey, *p* < 0.05)
